# Distribution of Extrasynaptic NMDA Receptors on Neurons

**DOI:** 10.1100/2012/267120

**Published:** 2012-04-30

**Authors:** Ronald S. Petralia

**Affiliations:** Advanced Imaging Core, NIDCD/NIH, 50 South Drive (50/4142), Bethesda, MD 20892-8027, USA

## Abstract

NMDA receptors are found in both synaptic and extrasynaptic locations on neurons. NMDA receptors also can be found on neurons in early stages prior to synaptogenesis, where they may be involved in migration and differentiation. Extrasynaptic NMDA receptors typically are associated with contacts with adjacent processes such as axons and glia. Extrasynaptic NMDA receptor clusters vary in size and may form associations with scaffolding proteins such as PSD-95 and SAP102. The best-characterized extrasynaptic NMDA receptors contain NR1 and NR2B subunits. Extrasynaptic NMDA receptors may be activated by glutamate spillover from synapses or from ectopic release of glutamate. Consequently, extrasynaptic NMDA receptor activation may occur under different circumstances than that for synaptic NMDA receptors, indicating different functional consequences for the neuron. In some cases, activation of extrasynaptic NMDA receptors may have a negative influence on the neuron, leading to cell damage and death, as may occur in some major diseases of the nervous system.

## 1. Introduction

Communication between neurons in the brain is the basis of learning and memory and of all actions and decisions. It involves secretion of a neurotransmitter from one neuron that activates the associated receptor on the next neuron, typically causing either an excitation or an inhibition of the response of that latter neuron. The most important and well studied of these is the excitatory neurotransmitter, glutamate. The main type of glutamate receptor (GluR) is the ionotropic GluR (iGluR), of which the activation by glutamate opens an ion channel in the receptor to pass sodium and potassium, and sometimes calcium [1]. Two other types of GluRs include those that lack a channel and activate a G protein (metabotropic GluRs or mGluRs) and a group of glutamate-gated chloride channels found only in some invertebrates [1, 2]. iGluRs probably evolved from structurally similar potassium channels in bacteria, after some of these acquired glutamate-binding domains for activation (GluR0s) [3]. iGluRs are found in both plants and animals, and in vertebrates include 2 major kinds—AMPA and NMDA (*N*-methyl-D-aspartate) receptors, and 2 relatively minor kinds—kainate and delta. AMPA receptors (AMPARs) are the major mediators of fast excitatory neurotransmission and pass sodium and potassium, but only certain subtypes can pass calcium. NMDA receptors (NMDARs) are major mediators of cell plasticity, that is, change in structure and function, resulting from their passing calcium into the neuron; this acts as a second messenger to activate specific cell mechanisms related to change of function of the neuron.

NMDARs are a type of iGluR that evolved with the first synapses in the simplest of eumetazoan animals, the cnidarians such as *Hydra*, and sea anemones [4–6]. The classic localization of NMDARs is within the postsynaptic density (PSD), which is a complex of scaffolding and signaling proteins. But localization of NMDARs is not dependent on synapses. The PSD is derived from a structure that precedes synapses and NMDARs, found in some form in the simplest animals known such as sponges and placozoans, and even in the unicellular ancestors of animals. The most prevalent components of the PSD and its precursor structure are the MAGUKs (membrane-associated guanylate kinases) and certain proteins with proline-rich domains, especially shank [4–7]. NMDARs can be bound into this PSD scaffold at the synapse, but they also can be localized in many other places on the surface of neurons; they also are found in many other kinds of cells, both in the nervous system and in other systems of the body (these will not be discussed in this paper). It is not always clear if these extrasynaptic NMDARs are free or are in close association with other proteins such as the MAGUKs [8, 9]. NMDARs, like other iGluRs, are made up of four subunits that together form the ion channel. Typically, the NMDAR contains 2 NR1 subunits, of which there are 8 splice variants, and 2 NR2 subunits, of which there are 4 kinds, NR2A-D. Less commonly, NMDARs can contain NR3 subunits (NR3A or NR3B); they can form as NR1/NR2/NR3, which exhibits low conductance and reduced calcium permeability, or NR1/NR3, which does not respond to glutamate, only to glycine or D-serine (i.e., glutamate binds to NR2 subunits, while glycine or D-serine binds to NR1 and NR3 subunits [10]). Interestingly, vertebrate NR2 subunits have C-terminal (cytoplasmic) domains that are five times larger than those of known invertebrate NR2 subunits, presumably indicating an increase in complexity of function of vertebrate NMDARs [4]; NR3 subunits also may be a unique development of vertebrates. In this paper, we will examine the distribution of the extrasynaptic NMDARs of neurons and discuss how they are arranged in comparison to synaptic NMDARs, and, to a lesser extent, we will discuss their function. Note that the extrasynaptic zone includes any part of the neuron outside of the synaptic active zone. It includes the perisynaptic zone, which is about 100 nm around the synapse; in this paper, when the perisynaptic is discussed, “extrasynaptic” will refer to the remaining extrasynaptic membrane of the neuron. Extrasynaptic NMDARs are there for one of two reasons: either they are located in an extrasynaptic site to serve a necessary function there that differs from the function of synaptic NMDARs, or they are in transit, either being stored temporarily or actively moving to synapses from sites of exocytosis, or from synapses to sites of endocytosis [11–13]. We will see examples of all of these possibilities as we explore extrasynaptic NMDAR distribution and function in dendrites prior to synaptogenesis, during postnatal development and in the mature neuron, and including those that lie close to the postsynaptic membrane and others that are found at sites of ectopic release of neurotransmitter.

## 2. NMDARs Prior to Synaptogenesis

 It is not surprising that extrasynaptic NMDARs are found extensively on young developing neurons prior to or during the formation of synapses; that is, NMDARs would be expected to reach extrasynaptic sites during the process of becoming arranged at synapses [8]. In cultured neurons from the visual cortex, prior to synapse formation (3-4 days *in vitro* [DIV]), clusters of NMDARs are transported in the dendrite cytoplasm in association with the MAGUK, SAP102, and, an early endosome protein, EEA1 [16]. These clusters are exocytosed to the dendrite surface using a SNAP23-mediated mechanism (see also Suh et al. [17]), and many of them cycle periodically between the cytoplasm and dendrite surface, as they pass down the length of the dendrite, until they eventually may be recruited to newly forming synapses [18]. Synaptogenesis precedes the acquisition of NMDARs to the new synapse and may begin with an accumulation of an adhesion protein such as NCAM or cadherin [19–21], but this will not be discussed in this paper.

**Figure 1:**
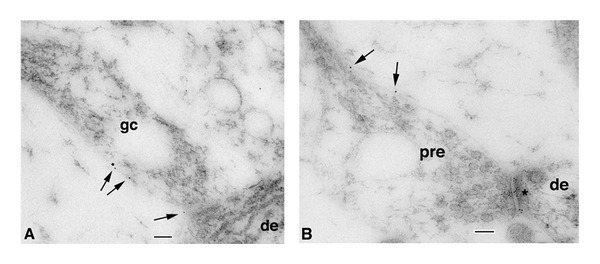
Immunogold localization of NR1 at the surface (5 nm gold particles indicated by arrows) of axonal growth cone (A; gc) and subsequent presynaptic terminal contact (B; pre) onto dendrites (de) in the CA1 stratum radiatum (A) or stratum oriens (B) of the postnatal day 2 (P2) hippocampus. Growth cones show characteristic collections of large and small endosomal vesicles. A is an axonal growth cone with a few obscure synaptic vesicles near the presynaptic contact, whereas B shows a better developed synapse, including a presynaptic terminal with numerous, distinct synaptic vesicles and high gold labeling on the postsynaptic membrane (asterisk: also, a clathrin-coated pit/vesicle is evident in the dendrite on the other side of the asterisk). The left part of the presynaptic terminal is expanded into a growth cone structure. Scale bars are 100 nm. Reprinted from part of Figure 2 from Wang et al. [14].

 Many of the NMDARs that reach the surface of the neuron prior to synapse formation are crucial to neuronal development; that is, they are not at the surface solely for transport to forming synapses. Indeed, these surface NMDARs are involved in many steps of neuronal migration and differentiation (reviewed in Wang et al. [14]) and even in neuroblast survival [22]. For example, NMDARs are crucial for dendritic arbor growth in optical tectal neurons from tadpoles of the amphibian *Xenopus *[23]. NR1/NR2B NMDARs are crucial for neuritogenesis and fasciculation of young neurons [24], and this is consistent with the prevalence of NR2B-containing NMDARs in early postnatal synapses [20, 21]. In fact, NMDARs containing NR1 and NR2B subunits are highly expressed in axonal growth cones (Figure 1) [14, 25, 26]. Indeed, these growth cone NMDARs mediate calcium influx [14], and calcium influx through growth cones can regulate growth cone turning [27] and the growth rate and branching of axons [28]. In the developing cerebellum, immature granule cells from the external germinal layer migrate along the processes of Bergmann glia to the internal granule layer (IGL). This migration is mediated by their surface NMDARs that are activated via a combination of glutamate (probably from more mature granule cells in the IGL) and D-serine (believed to be a necessary coagonist of NMDARs) released by the Bergmann glia [29, 30]. A curious parallel to these phenomena of NMDAR-mediated migration of growth cones and neurons is found in plants that have a kind of iGluR closely related to those found in animals [3]. These iGluRs form calcium channels on the tips of growing pollen tubes that respond to D-serine released by the pistil (i.e., the female organ that the pollen tubes must contact for fertilization), and this D-serine consequently controls pollen tube growth [31]. These plant iGluRs are not NMDARs and require only the D-serine as an agonist; in another interesting parallel, some vertebrate NMDARs may be activated by D-serine alone without glutamate as a coagonist (neuropeptide-releasing axon terminals [32]; NR1/NR3 NMDARs on myelin [33]).

## 3. Postnatal Development

While the distribution of extrasynaptic NMDARs has been little studied *in vivo* in the brain [8], it has been studied more fully in the development of neurons *in vitro*. Physiological studies indicate that about 75% of NMDARs are extrasynaptic at ~1 week *in vitro* (WIV) [37–39], while maybe 80–90% are extrasynaptic based on immunocytochemical studies. By 2 WIV, physiological studies then show the levels decreasing to 20–50% [40]. It is difficult to know the exact correlation of these levels *in vivo*, but the hippocampal slice study of Harris and Pettit [41] indicates that about 36% of NMDARs are extrasynaptic at P14-21.

During postnatal development, NR1 is always prevalent, while NR2B, NR2D, and NR3A are high early and decrease with age (especially the latter two), and NR2A, NR2C, and NR3B are low early and increase dramatically during later postnatal development [10, 42]. The expression of NR1 splice variants containing the N1 cassette (exon 5) may increase in extrasynaptic NMDARs in cerebellar granule cells during postnatal development [43]. NR3B is widely distributed in the brain but extrasynaptic locations have not been examined yet. NR2D-containing NMDARs may be exclusively extrasynaptic [44], but have not been well studied. NR2C-containing NMDARs also have not been well studied but probably are found in both synaptic and extrasynaptic locations [45, 46]. NR2A and NR2B have been studied much more thoroughly; it is thought generally that during postnatal development, extrasynaptic NMDARs contain mainly NR2B, but not NR2A, with adult neurons having mainly NR2B in extrasynaptic NMDARs and mainly NR2A in synaptic NMDARs. Biochemical studies of the hippocampus indicate that NR2B is common in early postnatal development while NR2A is relatively rare, and this pattern reverses as development progresses; similarly, hippocampus synapses show an equivalent change in NR2B and NR2A levels in the same time periods [20, 47] (a similar trend has been described for synapses in the cerebral cortex and thalamus [48, 49]). Furthermore, immunocytochemical and physiological studies of neuronal cultures support the idea that in general extrasynaptic NMDARs contain mainly NR2B, while extrasynaptic NMDARs with NR2A decrease with age and synaptic NMDARs with NR2A become more important as the neuron matures [12, 13, 29, 38, 50, 51]. However, Harris and Pettit [41] found similar levels of functional NMDARs with NR2B in synaptic and extrasynaptic sites in P14-21 hippocampal slices, and Petralia et al. [8] found similar distributions of NR2A and NR2B in extrasynaptic sites at 2-3 WIV, suggesting that the proposed dichotomy of mainly synaptic NR2A and mainly extrasynaptic NR2B that occurs during postnatal development may not be so definitive. This will be discussed more below in terms of distribution at adult synapses. In addition to whole receptors, some extrasynaptic NR2B-containing NMDARs may remain functionally active on the surface following C-terminal cleavage of the NR2B by calpain [52].

NR3A-containing NMDARs are mainly present in early postnatal development as noted above and are prevalent in perisynaptic and extrasynaptic sites; also, within synapses, NR3A tends to be concentrated in the outer portion of the synapse [53]. Maturation of the synapse may involve the removal of NR3A-containing NMDARs from the synapse, facilitated by binding to the endocytic adaptor protein, PACSIN1, which could target these NMDARs to the perisynaptic zone where they could be endocytosed via clathrin-coated pits.

**Figure 2:**
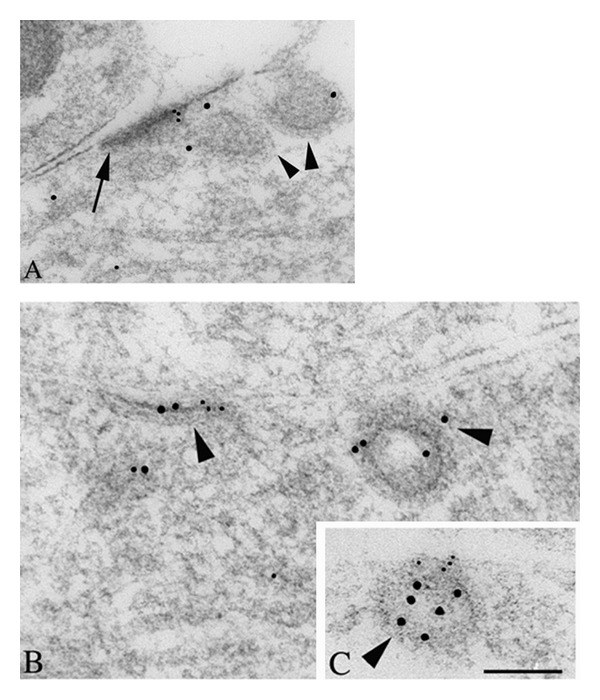
Double immunogold labeling of clathrin-coated pits/vesicles (CCP/V; arrowheads) associated with a “bare density” (A; arrow) and extrasynaptic membrane regions (B, C) in the CA1 stratum radiatum of the P2 hippocampus, labeled for NR1 (A, B; 5 nm gold) or NR2A/B (C; 5 nm gold), and clathrin (10 nm gold). The “bare density” (labeled for NR1) on the dendrite in A actually has a fairly close association with an adjacent process. In the dendrite in B, NR1 and clathrin label an early, flat CCP/V adjacent to a CCP/V that is pinching off. In C, NR2A/B labeling on the cell surface is continuous with a clathrin-labeled CCP/V. Scale bar is 100 nm. Reprinted from part of Figure 3 from Petralia et al. [15].

During postnatal development in hippocampal neurons *in vivo*, extrasynaptic NMDARs often are found in distinctive densities (Figure 2) [8, 15, 20, 47]. While some of these are relatively thin and may represent sites of new synapse formation, others are as thick as those of mature PSDs. These thicker ones also may contain some proteins commonly associated with synaptic NMDARs, including PSD-95, SAP102, and SynGAP; they are called “bare densities” and are believed to be remnants of former synapses. Preliminary studies of NMDAR distribution in neuron cultures using preembedding immunogold labeling show clusters of extrasynaptic NMDARs in “islands” associated with distinct cytoplasmic densities, with similar densities (i.e., this is a single-labeling method) labeling for other proteins including SAP102, GKAP, shank, and homer [54]. However, it is not clear if these latter densities seen *in vitro* are functionally the same as the “bare densities” seen in the postnatal brain. Extrasynaptic NMDARs in the postnatal brain also are associated with clathrin-coated pits, which are especially common in early development (Figure 2). These are sites of endocytosis of NMDARs, either for recycling (see previous section) or degradation [8, 15, 53].

## 4. Distribution in Relation to Sites of Synaptic and Ectopic Release of Glutamate

**Figure 3:**
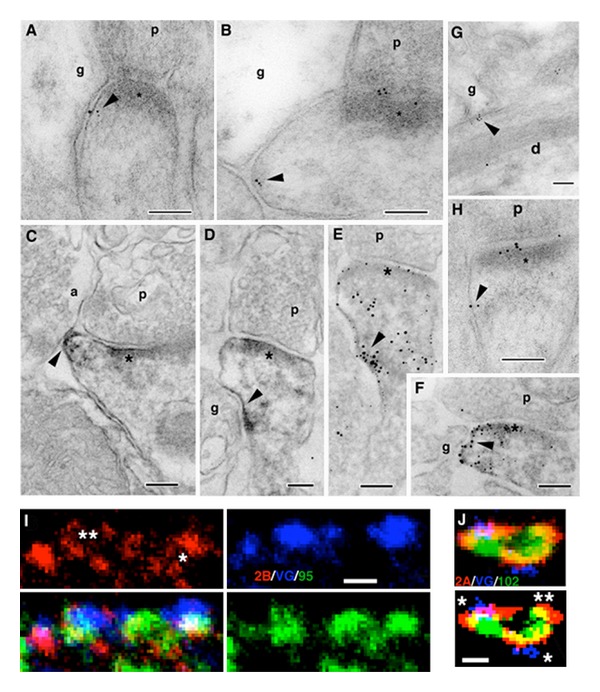
Extrasynaptic NMDARs (NR1 antibody; arrowheads) in the CA1 stratum radiatum of the adult hippocampus (A–H) using immunogold (AB,G,H) or EM immunoperoxidase/DAB (C–F: E and F were processed further with silver/gold toning), and immunofluorescence colocalization of NR2B/VGLUT1/PSD-95/93 (I) or NR2A/VGLUT1/SAP102 (J) in cultured hippocampal neurons. For A–H, extrasynaptic NMDARs can be seen on postsynaptic spines (A–F, H) or dendrites (G; d) adjacent to other processes, including glia (g) and various neuronal processes (see text for details). a: axon terminal; p: presynaptic terminal; asterisk: postsynaptic density. In I, NR2B labeling (red) forms in a perisynaptic ring (**) around synaptic PSD-95/93 (green) and the terminal (labeled with the presynaptic marker, VGLUT1 [blue]) and forms a ring of three puncta around an extrasynaptic punctum of PSD-95/93 (*). In J, this is an enlarged region found along a thin, distal dendrite. Note how NR2A labeling (red) is spread in the perisynaptic regions surrounding two synapses (*): SAP102 (green) forms around the enlargement in conjunction with both the synaptic and extrasynaptic (**) NR2A; the bottom image is a high contrast version of the top one. Scale bars are 100 nm for A–H and 500 nm for I, J. Reprinted from parts of Figures 2, 5, and 6 from Petralia et al. [8].

**Figure 4:**
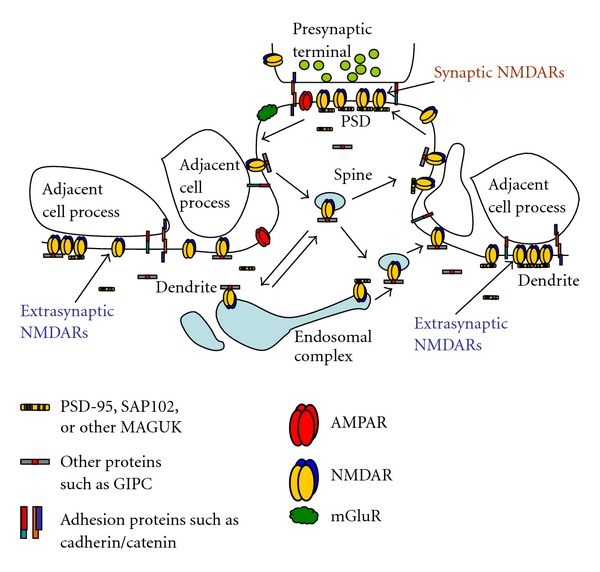
Diagram illustrating the synaptic and extrasynaptic distributions of NMDARs and associated scaffolding and adhesion proteins, and especially the associations of extrasynaptic NMDARs with adjacent cell processes (see text for details). Although not discussed in this paper, note that other GluRs (AMPARs, kainate and delta iGluRs, and metabotropic GluRs [mGluRs]) are found at synapses and in extrasynaptic locations. AMPARs are typically the most abundant GluRs at synapses and may also be more common than NMDARs in extrasynaptic locations in some neurons. mGluRs are also widespread; some forms are particularly abundant in the perisynaptic zone. Like NMDARs, these GluRs also show close associations with other proteins that affect their trafficking and localization (not illustrated here). The arrangement of different GluRs such as AMPARs and NMDARs within the synapse has been studied but still is not well defined. And although some AMPARs and NMDARs may traffic together in neurons in early development [18], little is known about the association of these different types of GluRs in extrasynaptic locations. For reviews, see Gereau and Swanson [1], Lu and Roche [34], and MacGillavry et al. [35]. Diagram modified from Figure 9 of Petralia et al. [8].

**Figure 5:**
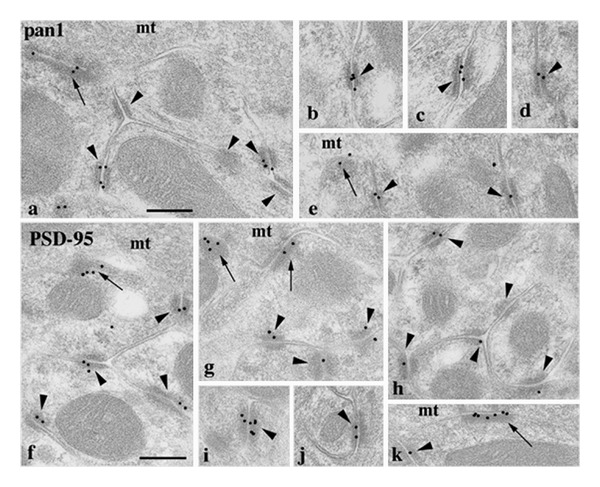
Localization of NMDAR (a–e) and PSD-95 (f–k) antibody labeling mossy terminal glomeruli in the cerebellar granular layer. The pan1 NMDAR antibody is shown here but this was corroborated using 3 more NR1 and 1 NR2A/B antibodies (see Petralia et al. [36] for details). Labeling is limited mainly to synapses (arrows: between the mossy terminal (mt) and dendrite processes from granule cells) and attachment plaques (arrowheads: mainly between granule cell dendrite processes). Scale bar is 200 nm. Reprinted from parts of Figures 2 and 3 from Petralia et al. [36].

As noted above, while many extrasynaptic NMDARs may be in transit to or from synapses, others appear to be in particular extrasynaptic sites for particular functions. In this section, we will explore the specific localization of extrasynaptic NMDARs. In adults, extrasynaptic NMDARs are distributed widely along the sides of the spine and along the dendrite surface [8, 55–57], as well as in presynaptic terminals. As evident both in culture and *in vivo*, most of these extrasynaptic sites are points of contact with an adjacent process including glia, axons and their terminals, as well as dendrites (Figures 3 and 4) [8, 58]. This is the normal state of the neuropil especially in the mature nervous tissue, and blank areas along a dendrite or spine surface are relatively rare. Furthermore, since NMDARs extend out about 10 nm from the surface of the membrane, these sites may contact proteins extending out from the adjacent cell membrane. Thus, such contact points could be functional; they could serve to anchor the NMDARs to a particular point, for example, to control the position of NMDARs relative to sites of glutamate release. Glutamate activation of extrasynaptic NMDARs can occur via spillover from adjacent synapses or via ectopic release from adjacent processes other than from synaptic active zones. Spillover from an adjacent active zone is especially important for activation of perisynaptic NMDARs [59–61]. The degree of activation of extrasynaptic NMDARs probably varies during development, due to changes in the size of extracellular space and level of expression of glutamate transporters [62, 63]. The extreme case of activation of extrasynaptic NMDARs via synaptic spillover is found in the cerebellar glomeruli (Figure 5) [36]. In this case, NMDARs are abundant in attachment plaques (puncta adherentia) between granule cell dendrites; these dendrites form synapses with the large central glutamatergic mossy terminal. NMDARs are concentrated at both these attachment plaques and synapses. In contrast, AMPARs are common only at the synapses. The mossy terminal glomerulus mostly excludes glial processes so that glutamate released from the synaptic active zones probably diffuses a great distance between the postsynaptic granule cell dendrites within the glomerulus [64] (reviewed by Petralia et al. [36] and Szapiro and Barbour [65]). NMDARs require a lower titer of glutamate to respond than do AMPARs, so that the NMDARs in the attachment plaques potentially could be exposed to sufficient glutamate, following high-level activation of the mossy terminal, to activate the NMDARs. Indeed, there is physiological evidence for extrasynaptic NMDARs here [66]. Excitation of these extrasynaptic NMDARs could cause plastic changes in the glomerulus as discussed below (see also discussions in Petralia et al. [36] and Rossi et al. [66]).

 Some extrasynaptic NMDARs could be activated by ectopic release of glutamate from axons, dendrites, or glia [67–69]. In the hippocampus, extrasynaptic NMDARs on dendrites and spines typically are apposed to glial or axonal processes as well as to other dendrites or spines, and thus many of these could be sites of ectopic release (Figures 3 and 4) [8]. There is evidence for the release of glutamate from glia to excite postsynaptic [69–71] and presynaptic [72] extrasynaptic NMDARs. In the latter case, astrocyte processes that contact the extrasynaptic sides of the presynaptic terminals of spine synapses of hippocampal granule cells are believed to release glutamate at these sites. The authors found “synaptic-like microvesicles” next to the glial membrane here, and immunogold labeling for NR2B on the presynaptic terminal extrasynaptic membrane in the same region. The authors believe that these are points of “focal communication,” separated spatially from the glutamate released from the active zone. This spatial precision allows the activation of these extrasynaptic NMDARs to more precisely control neurotransmitter release from the terminal. Ectopic release sites can occur from adjacent axons; ectopic release from white matter axons can activate glial NMDARs and AMPARs [65, 73–75] (see also a study on D-serine activation of glial NMDARs [33]).

 The size of extrasynaptic NMDAR clusters is difficult to determine [8]. Generally, both immunoEM and immunofluorescence studies indicate that synaptic regions tend to be larger than extrasynaptic NMDAR clusters in adult rats and mature neuron cultures. The smallest clusters seen with electron microscopy (EM) were a single 5 nm gold particle or a patch of immunoperoxidase labeling (generated by the DAB method) of 30–50 nm. The latter could represent a single molecule (spread wider due to a bleeding artifact of the DAB method) or maximally, a cluster of four NMDARs (NMDARs are about 20 nm in diameter [76]). ImmunoEM of transfected cultured neurons reveals extrasynaptic NMDAR clusters that tend to be larger than those of native extrasynaptic NMDAR clusters; related to this, Groc et al. [77] showed evidence that native and transfected NMDARs behave differently.

## 5. Proteins Associated with Extrasynaptic NMDARs

While some distinct density structures are associated with extrasynaptic NMDARs in early postnatal development as discussed above (Figure 2), these are not as distinct in the adult (Figure 3) [8]. The exception is the attachment plaques of the cerebellar glomeruli just discussed (Figure 5) [36]. Extrasynaptic NMDARs probably associate with a number of other proteins, even if the association is not arranged in a structure as complex as a PSD. Extrasynaptic NMDARs may be associated with various adhesion proteins (discussed in Petralia et al. [8]. Notably, Petralia et al. [8] showed colocalization at these sites with cadherin and catenin; these are important components of the PSD [20] and of attachment plaques of the cerebellar glomeruli (Figure 5) [36]. A number of PDZ proteins, involved in the trafficking and scaffolding of NMDARs, may associate with the NMDARs at these extrasynaptic sites. One of these, GIPC (GAIP [G alpha-interacting protein]-interacting protein, C-terminus) appears to be localized to extrasynaptic NMDARs preferentially (Figure 4) [78]. The MAGUK, betaSAP97, may preferentially localize NMDARs to extrasynaptic sites [79]. MAGUKs such as SAP102 and PSD-95 are associated with NMDARs at extrasynaptic sites (Figures 3 and 4) [8, 80], and especially the MAGUK SAP102 may be involved in the early trafficking of NMDARs to extrasynaptic and synaptic sites, as discussed above [16, 81, 82]. Both PSD-95 (Figure 5) [36] and SAP102 (R.S. Petralia, unpublished data) are found in the NMDAR-containing attachment plaques of the cerebellar glomeruli. PSD-95, SAP102, and the MAGUK-associated Ras-GTPase activating protein, SynGAP, are found in the “bare densities” seen in early postnatal development (Figure 2) [47] and these densities were shown to contain extrasynaptic NMDARs as discussed above.

## 6. Functions of Extrasynaptic NMDARs

There are two major broad group of effects attributed to extrasynaptic NMDARs: (1) positive effects on activity in the neuron or in the modulation and plasticity of an individual synapse and (2) more negative effects whereby activation of extrasynaptic NMDARs promotes neuron death; the latter is in contrast to promotion of neuron survival via activation of synaptic NDMARs. For both positive and negative cases, the effects are related to the physical location of the NMDARs on the neuron, the kinds of NMDARs, and to their association with different collections of various scaffolding and signaling proteins [83].

Related to the positive effects, we already have discussed functions of extrasynaptic NMDARs in early migration and growth of neurons and neuroblast survival (i.e., prior to or during formation of the first synapses). Several examples of these positive effects also have been described in postnatal and adult animals. Calcium elevation in astrocytes in the hippocampus can cause release of glutamate from these astrocytes, and this can excite multiple CA1 neurons synchronously via activation of NR1/NR2B-type extrasynaptic NMDARs; such synchronized activity may be central to information processing in the brain [70]. Also, as discussed above, Jourdain et al. [72] showed that glutamate released from some astrocytes in the hippocampus can activate presynaptic, NR2B-containing extrasynaptic NMDARs to enhance synaptic strength. In addition, release of glutamate from several synapses may converge on a shared population of NR2B-containing extrasynaptic NMDARs to monitor overall activity in the system [62, 84].

Within an individual synapse, release of glutamate from the presynaptic active zone can activate NMDARs both in the postsynaptic membrane and others in the perisynaptic/extrasynaptic membrane of the postsynaptic process, the latter accomplished by glutamate spillover as discussed above. In the basal dendrites of layer 5 pyramidal neurons in the mouse prefrontal cortex, glutamate spillover can promote the generation of NMDA spikes, and this probably increases the extent of synaptic plasticity that occurs along the dendrite [85]. In retinal ganglion cell synapses, extrasynaptic NMDARs only can be activated during evoked responses, since glutamate transporters prevent activation of extrasynaptic NMDARs by glutamate release from a single vesicle [86]. In a comparison of ON and OFF bipolar cell synapses on retinal ganglion cells, Zhang and Diamond [87] found NMDARs containing NR2A, the NR1C2' splice variant of NR1, and the MAGUKs PSD-95 and PSD-93 in the postsynaptic membrane/density of these synapses, and this was preferentially at OFF synapses. In contrast, perisynaptic NMDARs contained NR2B, the NR1C2 splice variant, and the MAGUK SAP102, and the perisynaptic NR2B at least was found preferentially at ON synapses. These different distributions of NMDARs may be related to the different dynamic ranges of light-evoked responses in ON and OFF retinal ganglion cells. In the spinal cord of the adult rat, some synapses of neurons from the substantia gelatinosa that may be involved in responses to pain contain synaptic non-NMDARs, NR2A-containing NMDARs, and extrasynaptic NMDARs containing NR2B (and NR2D less commonly); the authors discuss evidence suggesting that activation of these synaptic versus extrasynaptic NMDARs is involved in responses to acute versus chronic pain, respectively [88].

The nematode worm, *Caenorhabditis elegans*, has a kind of synapse where non-NMDARs and NMDARs are separated into synaptic and extrasynaptic zones, respectively [89]. In these worms, mechanical, osmotic, and chemical stimuli are detected by polymodal ASH neurons that subsequently release glutamate at synapses on interneurons to trigger avoidance responses. Whereas mechanical stimuli may cause the release of a small amount of glutamate and activate only the non-NMDARs (made up of subunits GLR-1 and GLR-2) found in the synapse, osmotic stimuli may induce the release of more glutamate that can spillover and thus activate both the synaptic non-NMDARs and extrasynaptic NMDARs. Interestingly, these synapses also contain glutamate-gated chloride channels (a special kind of GluR limited to some invertebrate groups, as noted in the Introduction) that may be involved in the reestablishment of forward movement following the avoidance response.

 One major mediator of synaptic plasticity is the adhesion protein combination of cadherin/catenin, which is present in synapses [90]. As noted above, cadherin/catenin also is associated with extrasynaptic NMDARs [8]. Cadherin along with other adhesion proteins called nectins helps regulate interneuron affinity and may be critical for the ordered association of axons and dendrites [91]. Cadherin adhesion is dependent on calcium, and perhaps extrasynaptic NMDARs help regulate local calcium levels to control cadherin adhesion as proposed for synapses [90]. In an early step in synaptic plasticity, calcium entry through NMDARs depletes the local synaptic environment of calcium and thus loosens the cadherin adhesion, allowing for the physical changes in the synaptic components involved in potentiation or depression of synapse function. This may also explain the curious high levels of extrasynaptic NMDARs at attachment plaques (which contain a large amount of cadherin) between granule cell dendrites in the cerebellar glomerulus, as discussed above (Figure 5) [36]. Both NMDARs and calcium entry may play roles in the plasticity associated with mossy fiber terminal-granule cell dendrite synapses [66, 92, 93]. But physical changes within the glomerulus that could accompany plasticity, assuming that the process is similar to what occurs in spine synapses, might be difficult; that is, the large number of attachment plaques should make the glomerulus a fairly rigid structure. Perhaps release of cadherin adhesion in these plaques, due to NMDAR activation, is important for glomerular plasticity.

While the examples discussed so far have focused on positive functions of extrasynaptic NMDARs, evidence is accumulating that activation of extrasynaptic NMDARs can promote cell death. In fact, several pathological conditions including ischemia and neurodegenerative diseases such as Huntington's, Alzheimer's, and Parkinson's may involve an abnormal increase in numbers of and/or signaling through extrasynaptic NMDARs (reviews [9, 83]). The pathways are complicated (see these reviews), but basically activation of synaptic NMDARs initiates a chain of reactions that have antioxidant and antiapoptotic effects, such as inactivating pro-death transcription factors including FOXO (forkhead box protein O) and p53 and activating pro-survival transcription factors such as CREB (cyclic-AMP response element binding protein). In contrast, preferential activation of extrasynaptic NMDARs produces prodeath effects such as CREB shut-off due to nuclear accumulation of Jacob (juxtasynaptic attractor of caldendrin on dendritic boutons protein), ERK1/2 (extracellular signal-regulated kinase 1/2) inactivation, FOXO activation, and calpain activation and subsequent STEP (striatal enriched tyrosine phosphatase) cleavage; the latter prevents STEP from inhibiting p38 MAP kinase, which can contribute to neuronal death. Pathological conditions can increase the expression or activation of extrasynaptic NMDARs and thus favor these prodeath pathways. For example, ischemia leads to reversed uptake of glutamate in astrocytes adjacent to extrasynaptic NMDARs on neurons, and ischemia induces DAPK (death-associated protein kinase) activation that then enhances extrasynaptic NMDAR function. In Huntington's disease, mutant huntingtin protein can redistribute synaptic NMDARs to extrasynaptic sites and can stabilize extrasynaptic NMDARs, for example, by increasing extrasynaptic NR2B binding to PSD-95.

Finally, NMDARs can show mechanosensitivity, and this may be involved in excitotoxicity following traumatic brain injury, due to the mechanosensitivity as well as to the increase in extracellular glutamate seen after this injury (discussed in Singh et al. [94]). In fact, NR1/NR2B NMDARs show significantly more stretch sensitivity than NR1/NR2A NMDARs; so it is possible that synaptic and extrasynaptic NR1/NR2B receptors could play a role in traumatic brain injury through this mechanism [94].

## 7. Conclusions and Future Directions

Extrasynaptic NMDARs include those that are in transit to and from synapses, and this can vary with development, and others that are located in extrasynaptic sites to perform functions different from those at synapses. We only are beginning to understand the arrangements and organization of extrasynaptic NMDARs and much more needs to be discovered [8, 12, 13]. We also need to know much more about how the differential distribution of NMDARs in synapses versus extrasynaptic areas can have different effects on the function of the neuron, both positive and negative. Finally, emerging knowledge of the role of extrasynaptic NMDARs in pathologies gives us hope to understand the etiology of major diseases of the nervous system [9, 83].
